# Improvement in Outcomes of Childhood Acute Myeloid Leukemia Treatment: A Survival Analysis at a Reference University Hospital in Rio de Janeiro, Brazil

**DOI:** 10.1002/cnr2.70640

**Published:** 2026-08-02

**Authors:** Thais Alcantara Bonilha, Deborah Sutter Ayres Pereira, Alice Maria Boulhosa de Azevedo, Adriana Martins de Sousa, Elaine Sobral da Costa, Marcelo Gerardin Poirot Land

**Affiliations:** ^1^ Gesteira Institute of Childcare and Pediatrics (IPPMG/UFRJ) Rio de Janeiro Brazil; ^2^ Internal Medicine Graduate Program UFRJ School of Medicine Rio de Janeiro Brazil; ^3^ National Science and Technology Institute for Children's Cancer Biology and Pediatric Oncology – INCT BioOncoPed Porto Alegre Brazil

**Keywords:** acute myeloid leukemia, acute promyelocytic leukemia, pediatrics, prognostic factors

## Abstract

**Background:**

Acute myeloid leukemia (AML) represents 15% to 20% of all pediatric acute leukemias and is responsible for nearly 30% of deaths in this population. While high‐income countries report overall survival (OS) rates near 75%, survival in Brazil remains significantly lower, ranging between 30% and 40%.

**Aims:**

The objective of this study is to analyze the probability of OS and event‐free survival (EFS) in pediatric patients with AML treated at a Brazilian university hospital, evaluate prognostic factors, and compare outcomes with international data.

**Methods and Results:**

This retrospective study included 51 patients (under 14 years old) diagnosed between 2004 and 2023 and treated with BFM‐based protocols. The median age was 4.6 years. Patients with acute promyelocytic leukemia (APL) achieved an OS of 100% and EFS of 90%. For other AML subtypes, the 5‐year OS and EFS were 53.7% and 43.7%, respectively. Implementation of improved supportive care (ISC) after 2008—including the opening of a pediatric ICU and better infection management—was a pivotal prognostic factor (*p* < 0.001). Following ISC implementation, OS increased dramatically from 8.3% to 74.5%, and the early death rate dropped to 5.7%, reaching levels comparable to high‐income countries.

**Conclusions:**

Pediatric AML outcomes in developing regions can achieve international standards when intensive chemotherapy is combined with optimized supportive care. The establishment of specialized pediatric intensive care and advanced management of infectious complications are essential pillars for bridging the survival gap in these populations.

**Abbreviations**
AML: Acute myeloid leukemia
APL: Acute promyelocytic leukemia
ATO: Arsenic trioxide
ATRA: All‐trans retinoic acid
BFM: Berlin–Frankfurt–Muenster
CNS: Central nervous system
EFS: Event‐free survival
HR: High risk
HSCT: Hematopoietic stem cell transplantation
IPPMG‐UFRJ: Martagão Gesteira Institute of Childcare and Pediatrics of the Federal University of Rio de Janeiro
IR: Intermediate risk
ISC: Improved supportive care
MRD: Minimal residual disease
OS: Overall survival
PICU: Pediatric Intensive Care Unit
SR: Standard risk
WBC: White blood cell

## 1 Introduction

Acute myeloid leukemia (AML) accounts for 15%–20% of all acute leukemias in children and is responsible for 30% of deaths in this population [1, 2]. Approximately seven cases per million per year are expected, and there is a bimodal peak of incidence, with the first peak in children under 2 years old and a second one in adolescents aged 15–20 years [1, 2].

In recent years, the cumulative dose of cytarabine has been progressively increased, and more effective anthracyclines, such as idarubicin or liposomal daunorubicin, have been introduced into the latest treatment protocols. In addition, hematopoietic stem cell transplantation (HSCT) has been introduced for the treatment of high‐risk or relapsed patients [3, 4, 5]. However, intensification of therapy is associated with increased toxicity and a high treatment‐related mortality rate [4]. Rasche et al. analyzed BFM group trials from 1987 to 2012, noting that while treatment intensification improved overall survival (OS), event‐free survival (EFS) remained stagnant. This discrepancy highlights the critical role of the group's rescue and supportive care in maintaining survival even after relapse [6].

In high‐income countries, intensive chemotherapy combined with improvements in supportive care has led to an increase in OS to nearly 75% [7, 8, 9]. In Brazil, there are few published studies, and the results are very different, with an OS rate of around 30%–40% [10, 11, 12, 13, 14].

The aim of this study was to analyze the probability of OS and EFS of pediatric patients with AML treated at the Martagão Gesteira Institute of Childcare and Pediatrics of the Federal University of Rio de Janeiro (IPPMG‐UFRJ), evaluate prognostic factors, and compare the results with those found in the literature.

## 2 Material and Methods

This retrospective survival analysis was performed on patients diagnosed between January 2004 and December 2023. Data were collected from the medical records of patients under 14 years of age with AML treated at IPPMG‐UFRJ in Brazil using AML BFM‐based protocols. All patients who received a *de novo* diagnosis of AML were included.

EFS was defined as the time from diagnosis to the date of the first event. Events considered were relapse, death, or secondary malignant neoplasm. The latter was identified based on SEER criteria, requiring distinct histological confirmation and the exclusion of late relapse or metastatic spread from the primary AML. OS was calculated from the date of diagnosis to the date of death from any cause. Deaths occurring within 42 days of diagnosis were considered early deaths, and those occurring within 15 days were considered very early deaths. Deaths in complete remission were classified as treatment‐related mortality.

The institution in this study experienced a significant improvement in supportive care (ISC), marked by the opening of the pediatric intensive care unit (ICU) in December 2007 and improvements in the early diagnosis and treatment of invasive fungal infections (e.g., galactomannan testing that started in 2008 and the use of voriconazole). Furthermore, in recent years, patients have had greater access to HSCT. To account for these changes, we created a variable named “ISC” and divided patients into two groups: those treated before the implementation of ISC (before 2008) and those treated after (2008 onwards).

Patients were stratified into risk groups according to the treatment protocol: standard (SR), intermediate (IR), and high (HR) risk. For analysis, patients were categorized into a “favorable” group if they were standard or intermediate risk, and an “unfavorable” group if they were high risk.

EFS and survival curves were estimated using the Kaplan–Meier method and compared using the log‐rank test. The Cox proportional hazards model was used to perform the multivariate analysis. Variables with *p*‐value < 0.2 in univariable analysis were selected for multivariable analysis. The proportional hazards (PH) assumption was checked using Schoenfeld residuals in R software. Only one variable did not satisfy the PH assumption—HSCT status (whether or not the patient underwent hematopoietic stem cell transplantation)—as it was a time‐dependent variable. Consequently, a time‐dependent adjustment was applied to this variable. After the assessment of the multicollinearity between the candidate variables, a parsimonious model was built through the backward elimination method, using the likelihood ratio test as the selection criterion.

Due to the small number of patients with promyelocytic leukemia and their high survival rate, univariate and multivariate analyses were restricted to the non‐M3 AML group.

Data were analyzed using software SPSS 30.0 and R software.

## 3 Results

A total of 51 patients were included in this study: 40 with non‐M3 AML and 11 with acute promyelocytic leukemia (APL/AML‐M3). Their clinical characteristics are summarized in Table 1. All patients were treated using a BFM‐based protocol. The median age at diagnosis was 4.6 years (range: 1 month–13 years), and the mean follow‐up period was 5.77 years (95% CI: 4.19–7.35).

**TABLE 1:** Clinical, laboratory, and genetic characteristics of pediatric patients with acute myeloid leucemia (*n* = 51).

Variable	All (%) (*n* = 51)	AML non‐M3 (%) (*n* = 40)	APL (%) (*n* = 11)	*p*
*Gender*				0.488
Female	18 (35.3)	13 (32.5)	5 (45.5)	
Male	33 (64.7)	27 (67.5)	6 (54.5)	
*Diagnosis*				< 0.001
AML M5	11 (21.6)	11 (27.5)	0	
AML M4	10 (19.6)	10 (25.0)	0	
AML M7	7 (13.7)	7 (7.5)	0	
AML M2	5 (9.8)	5 (12.5)	0	
AML M0	2 (3.9)	2 (5.0)	0	
AML NE	2 (3.9)	2 (5.0)	0	
AML M1	1 (2.0)	1 (2.5)	0	
AML M1/M2	1 (2.0)	1 (2.5)	0	
AML M4eo	1 (2.0)	1 (2.5)	0	
APL	11 (21.6)	0	11 (100.0)	
*CNS Status*				0.429
Positive	1 (2.0)	1 (2.5)	0	
Negative	40 (78.4)	30 (75.0)	10 (90.9)	
Missing	10 (19.6)	9 (22.5)	1 (9.1)	
*Protocol*				0.468
BFM 1998	10 (19.6)	9 (22.5)	1 (9.1)	
BFM 2004	13 (25.5)	9 (22.5)	4 (36.4)	
BFM 2012	26 (51.0)	20 (50.0)	6 (54.5)	
MLDS 2006	2 (3.9)	2 (5.0)	0	
*Initial WBC*				0.055
≦ 50 000	32 (64.0)	23 (57.5)	9 (90.0)	
> 50 000 to ≦ 100 000	6 (12.0)	6 (15.0)	0	
> 100 000 to ≦ 150 000	3 (6.0)	3 (7.5)	0	
> 150 000 to ≦ 200 000	2 (4.0)	1 (2.5)	1 (10.0)	
> 200 000	7 (14.0)	7 (17.5)	0	
*Initial platelet count*				0.573
≧ 70 000	15 (29.4)	11 (27.5)	4 (36.4)	
< 70 000	36 (70.6)	29 (72.5)	7 (63.6)	
*Risk group*				< 0.001
SR	18 (35.3)	9 (22.5)	9 (81.8)	
IR	7 (13.7)	7 (17.5)	0	
HR	26 (51.0)	24 (60.0)	2 (18.2)	
*Cytogenetics (n = 33)*				0.012
Complex karyotype	1 (3.0)	1 (3.8)	0	
del [11]	2 (6.1)	2 (7.7)	0	
Inv 16	3 (9.1)	3 (11.5)	0	
*t*(8;21)	2 (6.1)	2 (7.7)	0	
*t*(15;17)	7 (21.2)	0	7 (100.0)	
Normal	5 (15.2)	6 (19.2)	0	
Others	13 (39.3)			
*Molecular biology (n = 29)*				< 0.001
AML::ETO	2 (6.9)	2 (9.1)	0	
inv 16	3 (10.3)	3 (13.6)	0	
FLT3 mutation	5 (17.2)	5 (22.7)	0	
PML::RARA	7 (24.1)	0	7 (100.0)	
Negative	12 (41.4)	12 (54.5)	0	

*Note:* Data are presented as absolute numbers and percentages *n* (%). The *p*‐values refer to the comparisons between the AML non‐M3 and APL groups using Qui‐Square test, with statistical significance defined as *p* < 0.05. Cytogenetic and molecular analyses were available only in subsets of patients.

Abbreviations: AML, acute myeloid leukemia; APL, acute promyelocytic leukemia (FAB M3 subtype); BFM, Berlin–Frankfurt–Münster; CNS, central nervous system status at diagnosis; SR/IR/HR, standard risk/intermediate risk/high risk; WBC, white blood cell count (expressed in cells/μL).

**FIGURE 1:**
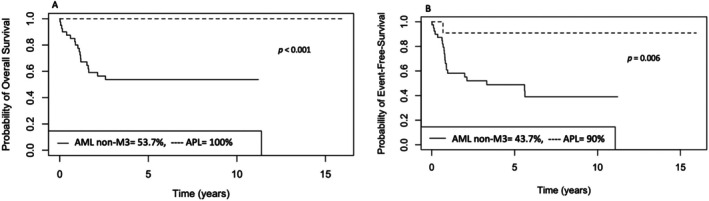
Overall Survival (OS) and Event‐Free Survival (EFS) of pediatric patients with AML. Kaplan–Meier estimates for OS (A) and EFS (B) for the entire cohort (2004–2023) stratified by AML subtype (*n* = 40 AML non M3 and *n* = 11 APL).

Patients with APL had a 5‐year probability of overall survival (pOS) and event‐free survival (pEFS) of 100% and 90%, respectively (Figure 1). Only one patient experienced a molecular relapse. Outcomes for patients with other AML subtypes were poorer: the 5‐year pOS and pEFS were 53.7% and 43.7%, respectively (Figure 1).

There were 4 (7.8%) early deaths: 1 (2%) occurring within 15 days and 3 (5.9%) between 16 and 42 days. Only 2 (4%) deaths occurred during the first complete remission. A total of 19 patients relapsed, 12 of whom died following the relapse. One patient (2%) presented with a secondary malignancy (ALL). Twelve patients (23.5%) proceeded to HSCT: three during the first complete remission and nine after relapse. Eight (66.6%) of these patients are currently alive.

In patients with non‐M3 AML, no statistically significant differences were found when analyzing 5‐year pOS and pEFS by sex, initial white blood cell (WBC) count, initial platelet count, or minimal residual disease (MRD) results after the first induction (Tables 2 and 3).

**TABLE 2:** Univariable overall survival analysis.

Category	*n*	HR	95% CI	5y‐OS	*p* (Log‐Rank)
*Gender*					
Female	13	1.00	1.00	53.8%	
Male	27	1.016	0.381–2.708	53.7%	0.975
*WBC (per μL)*					
< 30 000	17	1.00	1.00	57.0%	
≧ 30 000	23	1.208	0.468–3.119	51.0%	0.695
*Platelet count (per μL)*					
≧ 70 000	11	1.00	1.00	63.6%	
< 70 000	29	1.583	0.521–4.812	49.8%	0.414
*Risk group*					
Favorable (SR/IR)	16	1.00	1.00	74.0%	
Unfavorable (HR)	24	2.934	0.960–8.967	40.6%	0.048
*MRD pos induction*					
Negative (< 0.1%)	4	1.00	1.00	75.0%	
Positive (≧ 0.1%)	20	0.722	0.080–6.470	78.5%	0.770
ttHSCT*					
No	28	1.00	1.00	49.0%	
Yes	12	0.815	0.613–1.086	64.2%	0.100
*Improvement in supportive care*					
No	12	1.00	1.00	8.3%	
Yes	28	0.182	0.069–0.480	74.5%	< 0.001
*Relapse*					
No	22	1.00	1.00	72.1%	
Yes	18	1.597	0.976–2.614	33.3%	0.054

*Note:* ttHSCT*: Evaluated as a time‐dependent covariate or status because did not satisfy the PH assumption.

Abbreviations: 5y‐OS, 5‐year overall survival; HR, hazard ratio (for Risk Group, HR refers to High Risk); MRD, minimal residual disease; SR/IR, standard risk/intermediate risk; ttHSCT, hematopoietic stem cell transplantation; WBC, white blood cell count.

**TABLE 3:** Univariable event‐free survival analysis.

Category	*n*	HR	95% CI	5y‐EFS	*p* (Log‐Rank)
*Gender*					
Female	13	1.00	1.00	53.8%	
Male	27	1.399	0.578–3.389	38.4%	0.455
*WBC (per μL)*					
< 30 000	17	1.00	1.00	50.5%	
≧ 30 000	23	1.489	0.649–3.416	38.6%	0.344
*Platelet count (per μL)*					
≧ 70 000	29	1.00	1.00	47.3%	
< 70 000	11	1.047	0.433–2.530	36.4%	0.919
*Risk group*					
Favorable (SR/IR)	16	1.00	1.00	67.7%	
Unfavorable (HR)	24	3.145	1.226–8.065	28.1%	0.012
*MRD pos induction*					
Negative (< 0.1%)	4	1.00	1.00	37.5%	
Positive (≧ 0.1%)	20	0.484	0.128–1.830	63.5%	0.274
ttHSCT*					
No	12	1.00	1.00	49.5%	
Yes	28	1.069	0.915–1.248	30.0%	0.400
*Improvement in supportive care*					
No	12	1.00	1.00	8.3%	
Yes	28	0.255	0.106–0.610	59.3%	0.001

*Note:* ttHSCT*: Evaluated as a time‐dependent covariate or status because did not satisfy the PH assumption.

Abbreviations: 5y‐EFS, 5‐year event‐free survival; HR, hazard ratio (for risk group, HR refers to high risk); MRD, minimal residual disease; SR/IR, standard risk/intermediate risk; ttHSCT, hematopoietic stem cell transplantation; WBC, white blood cell count.

Three variables showed prognostic impact and were statistically significant in the univariable analysis: risk group, HSCT status, and ISC. These variables were selected for multivariable analysis.

After stratifying patients by risk group, outcomes in the favorable group (SR/IR) were significantly better than in the unfavorable group (HR): pOS and pEFS were 74% and 67.7% versus 40.6% and 28.1% (*p* = 0.048 and 0.012, respectively) (Tables 2 and 3).

**FIGURE 2:**
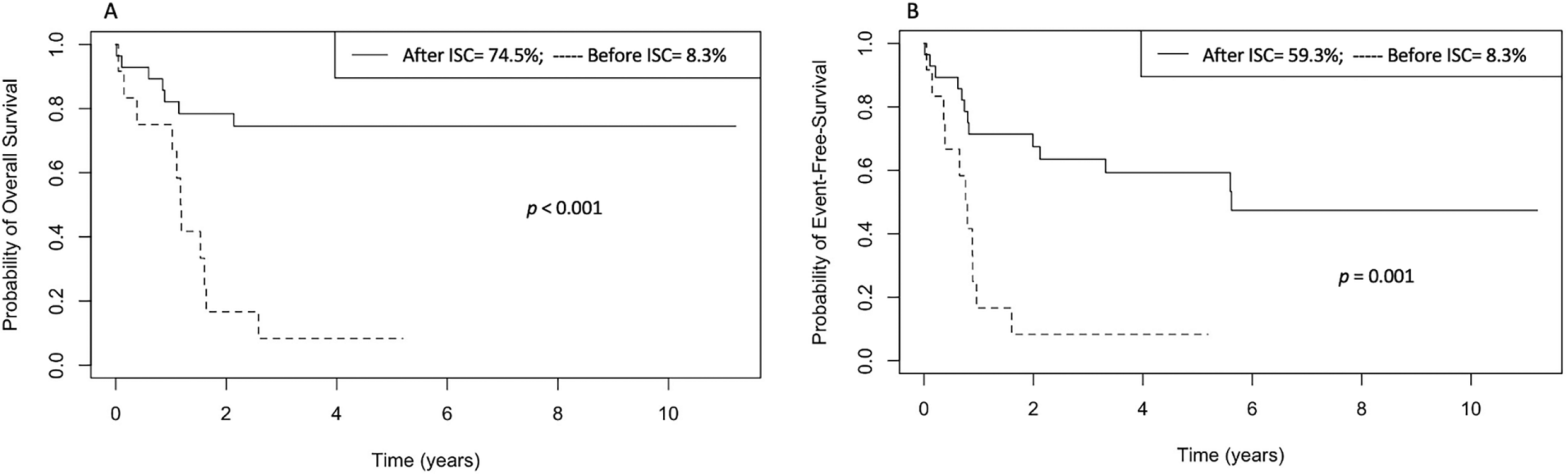
Impact of Improvement in supportive care (ISC) on patient outcomes. Comparison of 5‐year overall survival (A) and 5‐year event‐free survival (B) between the period prior to 2008 (*n* = 12) and the period following the implementation of enhanced supportive care protocols and specialized pediatric intensive care (*n* = 28).

When comparing the pOS and pEFS before (*n* = 12 patients) and after (*n* = 28 patients) the implementation of ISC, a significant difference in outcomes was observed (before ISC: 8.3% and 8.3%; after ISC: 74.5% and 59.3% (*p* < 0.001 and *p* = 0.001), respectively) (Figure 2).

The HSCT variable was adjusted for time; pOS and pEFS for patients who underwent HSCT were 64.2% and 30%, compared to 49% and 49.5% for those who did not (*p* = 0.1 and 0.4, respectively) (Tables 2 and 3).

For the multivariable analysis, three variables with significance at the *p* < 0.2 (ISC, ttHSCT and risk group) were selected for the inicial model. In the OS analysis, only ttHSCT and ISC remained in the final model, whereas in the EFS analysis, only risk group and ISC remained. Ultimately, only ISC was statistically significant (*p* < 0.05) with a hazard ratio (HR) of 0.175 (95% CI: 0.065–0.469) for OS and 0.359 (95% CI: 0.138–0.934) for EFS (Table 4).

**TABLE 4:** Multivariable analysis for overall survival and event‐free survival.

Category	HR	Overall survival	*p*	HR	Event‐free survival	*p*
		95% CI			95% CI	
*Risk group*						
SR/IR	—	—	—	1.00		
HR	—	—	—	2.137	0.747–6.112	0.157
ttHSCT*						
No	1.00			—	—	—
Yes	0.805	0.602–1.076	0.143	—	—	—
*Improvement in supportive care*						
No	1.00			1.00		
Yes	0.175	0.065–0.469	< 0.001	0.359	0.138–0.934	0.036

*Note:* ttHSCT*: Evaluated as a time‐dependent covariate or status because did not satisfy the PH assumption. For the multivariable analysis, 3 variables (ISC, ttHSCT and risk group) with *p* < 0.2 were select for the inicial model. In the OS analysis only ttHSCT and ISC remained in the final model and in the EFS analysis only risk group and ISC remained.

Abbreviations: CI, confidence interval; HR, hazard ratio (for risk group, HR refers to high risk); ttHSCT, hematopoietic stem cell transplantation; SR/IR, standard risk/intermediate risk; “–” means that the variable was not present in the final model.

## 4 Discussion

This study evaluated outcomes and risk factors for mortality in 51 patients with AML treated over a 20‐year period at a single institution. Our cohort exhibited a distinct profile compared to international literature, with a median age of 4.6 years—deviating from the classic bimodal peak incidence. We also observed a higher male predominance, with a 1.8:1.0 ratio. In contrast, the AML‐BFM 93 and 98 protocols reported a higher proportion of adolescents (42% > 10 years vs. 20% in our study), while the AML‐BFM 2012 trial showed an older median age (7.79 years) and a more balanced sex ratio (1.1:1.0) [4, 15]. Similar older age trends were documented in Kenya (42.5% > 10 years and a median age of 8.6 years) [16].

Within Brazil, where studies remain scarce, Lins et al. (Recife) reported a more comparable profile, with a median age of 6.4 years (excluding APL) and a 1.7:1.0 sex ratio [13]. Additionally, a multicenter study from Southern Brazil found an older population (10.5 years) but maintained a sex ratio consistent with our data (1.7:1.0) [14].

The lower proportion of adolescents in our cohort may have contributed to the favorable outcomes observed, as age over 10 years is a recognized risk factor for early and treatment‐related mortality [4]. Furthermore, a population‐based study from the United States using the Surveillance, Epidemiology, and End Results (SEER) database found that patients aged 10 to 19 years at diagnosis had a higher risk of mortality [HR 1.23; 95% CI: 1.12–1.36] [17].

In our cohort, overall rates of early death (7.8%) and mortality during first complete remission (CR1, 4%) were lower than those of other Brazilian centers, though they still exceed the benchmarks of leading international institutions. Notably, following ISC implementation, these figures improved significantly: the early death rate fell to 5.7% and CR1 mortality reached zero. These outcomes align with results from high‐income countries; for context, the BFM group documented a 6% early death rate in their 2004 and 2012 trials, with CR1 mortality between 1.5% and 5% [18]. This performance stands in sharp contrast to other domestic studies that show substantially higher mortality, such as the 11.8% early death and 9.5% CR1 mortality observed at IMIP [13]. Similarly, treatment‐related complications accounted for 12.5% of deaths in Southern Brazil [14], while a historical study conducted in the state of Minas Gerais recorded an early death rate as high as 28.9% [11].

Analysis of the causes of early death and treatment‐related mortality—which occurred predominantly due to infection or hemorrhage—underscores the vital importance of robust supportive care in the management of pediatric AML, a finding consistent across all reviewed studies.

Patients with AML often present at admission in critical clinical condition due to the high disease burden, facing significant risks of hemorrhage, infection, tumor lysis syndrome, or leukostasis. This is particularly prevalent in cases of acute promyelocytic leukemia (APL) and monoblastic leukemia [4, 9, 19]. Furthermore, the administration of intensive chemotherapy regimens results in prolonged periods of severe myelosuppression, which substantially increases the risk of life‐threatening infections [20, 21, 22]. Consequently, institutions managing these patients must provide specialized multidisciplinary teams, ICUs, and access to high‐complexity diagnostic tools and advanced therapeutics to effectively manage these complications.

The consolidation of ISC as a pivotal determinant of survival in our cohort reflects the successful mitigation of historical structural, logistical, and therapeutic challenges inherent to pediatric AML management. First, the establishment of a specialized pediatric intensive care unit (PICU) necessitated a comprehensive clinical restructuring that transcended the mere acquisition of technological infrastructure; it demanded the continuous training of a multidisciplinary team adept at the immediate recognition of specific oncological emergencies and the aggressive management of septic shock within critical time windows. Second, the management of invasive fungal infections (IFIs)—historically associated with dismal outcomes due to the dose‐limiting toxicities of conventional amphotericin B deoxycholate and the low sensitivity of standard culture‐based diagnostics—was revolutionized by a transition toward a proactive, preemptive strategy. This paradigm shift was driven by the integration of early high‐resolution computed tomography (HRCT) scanning and non‐invasive biomarkers (such as galactomannan assays), which facilitated targeted, timely intervention with modern broad‐spectrum antifungal agents characterized by favorable toxicity profiles, including liposomal formulations and echinocandins. Synergistically, advanced infectious surveillance and high‐complexity critical care provided an indispensable clinical safety net that minimized treatment‐related mortality, ultimately enabling patients to safely adhere to and complete the intensive chemotherapy regimens essential for long‐term disease control.

The significant impact of these changes is evident, as the survival rates following supportive care improvements became comparable to those of leading international treatment centers, with a 5‐year OS (OS) of 74.5% and an EFS of 59.3% [6]. In comparison, the main international research groups in pediatric hematology report OS rates ranging from 69% to 77.7% and EFS rates of 53% to 66.7% [5, 23]. Throughout this period, minimal changes were introduced to the first or second‐line therapeutic protocols. The AML‐BFM 2004 and 2012 regimens featured largely identical chemotherapy backbones, with the notable exception of acute promyelocytic leukemia (APL) management. Instead, the most substantial modifications pertained to risk stratification strategies. Crucially, neither protocol utilized MRD monitoring for risk‐adapted stratification.

An improvement in the rescue of relapsed patients has been observed over the years, with a higher number of patients undergoing HSCT and achieving long‐term survival after relapse. Stankiewicz et al. reported a 5‐year OS of 48.9% for patients who underwent HSCT in Poland, compared to 25% for those treated without transplantation [24]. Similarly, Vedi et al., in a study spanning Australia and New Zealand, demonstrated the critical role of HSCT in second complete remission (CR2), reporting a 50% survival rate among transplant recipients versus 12% for those treated with chemotherapy alone (*p* < 0.001) [25]. In our cohort, the impact of HSCT on OS did not reach statistical significance (5‐year OS of 64.2% for the HSCT group vs. 49% for the non‐HSCT group; *p* = 0.1), likely due to the limited sample size. Nevertheless, a stark contrast in outcomes was observed when comparing patients who underwent HSCT before and after 2010. Prior to 2010, all patients received transplants following relapse and subsequently died due to disease recurrence. Conversely, after 2010, 33% of patients underwent transplantation in CR1 and 67% in CR2, with only one death recorded following the procedure.

In our cohort, we found no significant difference in outcomes between children with positive and negative MRD after induction, a finding that contrasts with the current literature. This lack of statistical significance may be attributed to the limited sample size of our study and should not be interpreted as evidence of no prognostic effect. The literature has already widely proven the prognostic importance of a positive MRD after induction. The BFM group demonstrated in 2015 the critical importance of achieving MRD negativity after the first induction cycle. Their data showed a significant statistical difference in 3‐year EFS between MRD‐negative and MRD‐positive children (71% vs. 48%, *p* = 0.029) [26]. Furthermore, a 2020 meta‐analysis of 81 publications suggests that MRD status remains a key prognostic factor in AML and may serve as a valid surrogate marker for both OS and disease‐free survival (DFS) in clinical trials [27].

Regarding risk stratification, our cohort demonstrated a remarkably high prevalence of high‐risk disease, with 60% of patients categorized into the unfavorable risk group (HR) and only 40% in the favorable group (SR/IR). This distribution contrasts sharply with findings from major international cooperative groups, such as the AML‐BFM and Children's Oncology Group (COG) trials, where the majority of pediatric AML patients (approximately 75% to 85%) are classified into standard or intermediate‐risk groups, and only 15% to 25% are defined as high risk [4, 5, 8]. This divergence in risk group distribution may be attributed to this limited sample size of our cohort or, alternatively, to a distinct molecular‐genetic profile unique to our population. However, this hypothesis cannot be definitively validated due to the historical scarcity of comprehensive cytogenetic and molecular data. Presently, recent advancements in diagnostic infrastructure and the routine implementation of these assays at our center allow for a more precise patient characterization, which will facilitate a deeper understanding of the regional biological landscape of pediatric AML in future studies. APL is a distinct entity among acute myeloid malignancies, typically characterized by the translocation t(15;17)(q24.1;q21.2) and the resulting *PML::RARA* fusion gene [28]. In our study, 11 patients (21.5%) were diagnosed with APL. This frequency is higher than that reported in the United States, where it accounts for 5%–10% of AML cases [29, 30]. In line with our observations, the literature indicates an increased prevalence of APL among Latin American, Southern European, and African populations, accounting for 17%–58% of pediatric AML cases [31]. Locally, Brazilian research has identified frequencies of 23.1% and 27.5%, further corroborating our results [32, 33].

In recent decades, the outcomes for patients with APL have improved substantially following the introduction of all‐trans retinoic acid (ATRA) and arsenic trioxide (ATO) [32, 34]. Nevertheless, these patients remain at high risk for early mortality, primarily due to hemorrhagic complications and/or thrombosis [35]. In our cohort, no deaths were observed in this subgroup, including early deaths; however, this should be interpreted with caution due to the small sample size. For comparison, a study from the Boldrini Children's Center reported an early death rate of 13.1%, with 83% of these cases attributable to thromboembolic events [32]. Another Brazilian study reported an early death rate of 6% in this population [33].

The results of this study demonstrate that pediatric AML outcomes approaching international standards may be achievable in specialized referral centers in resource‐limited settings when intensive chemotherapy is combined with optimized supportive care. The implementation of the ISC and the pediatric ICU was a pivotal factor in increasing OS from 8.3% to 74.5%. This study demonstrates that survival rates have reached excellent levels in the last years, corroborating that the risk‐group stratification and specialized supportive care remain the fundamental pillars for improving patient prognosis.

Several limitations inherent to this study must be acknowledged. First, its retrospective observational design conducted at a single center may restrict the generalizability of our findings to other institutional settings or regions within Brazil. Second, the relatively small sample size (*n* = 51) distributed over a long study period spanning two decades (2004–2023) limited the statistical power of our multivariate and subgroup analyses. Third, due to the historical nature of the data, cytogenetic, molecular, and MRD records were incomplete for some patients, reflecting the progressive evolution of diagnostic capabilities at our center over time. Furthermore, the complex and multifaceted nature of ISC—which concurrently involved the opening of a specialized pediatric ICU, enhanced management of infectious complications, and broader access to HSCT—makes it challenging to isolate the independent impact of each individual intervention. Despite these constraints, this study provides valuable, long‐term, real‐world evidence regarding the critical role of supportive infrastructure in improving pediatric AML outcomes in resource‐limited scenarios.

## Author Contributions

**Thais Alcantara Bonilha:** conceptualization, investigation, writing – original draft, methodology, formal analysis, data curation. **Marcelo Gerardin Poirot Land:** conceptualization, investigation, validation, writing – review and editing, formal analysis, data curation, supervision, project administration. **Elaine Sobral da Costa:** conceptualization, visualization, writing – review and editing, supervision. **Adriana Martins de Sousa:** investigation, visualization. **Deborah Sutter Ayres Pereira:** conceptualization, investigation, writing – original draft, methodology, formal analysis, data curation. **Alice Maria Boulhosa de Azevedo:** investigation, visualization.

## Funding

This research was financed in part by the Coordenação de Aperfeiçoamento de Pessoal de Nivel Superior—Brazil (CAPES) – Finance Code 001. This work was supported by the Conselho Nacional de Desenvolvimento Cientifico e Tecnologico (CNPq, MCTI, Brazil) grant number 406484/2022–8 (INCT BioOncoPed).

## Ethics Statement

The present study was approved by IPPMG‐UFRJ Ethics Committee on March 26, 2024 under the registration number 6.726.041. Since this was a retrospective study that did not involve any intervention, the requirement for informed consent was waived.

## Conflicts of Interest

The authors declare no conflicts of interest.

## Data Availability

The data that support the findings of this study are available from the corresponding author upon reasonable request.
